# Application of Orthopedic Dual Sliding Compression Plate (ODSCP) in High Medial Tibial Open Wedge Osteotomies

**DOI:** 10.5812/ircmj.8371

**Published:** 2013-04-05

**Authors:** Seyed Salman Samani, Amir Reza Kachooei, Mohammad Hosein Ebrahimzadeh, Farzad Omidi Kashani, Reza Mahdavian Naghashzargar, Shiva Razi

**Affiliations:** 1 Orthopedic and Trauma Research Center, Mashhad University of Medical Sciences, Mashhad, IR, Iran

**Keywords:** Osteotomy, Range of Motion, Articular, Genu Varum

## Abstract

**Background:**

Angular deformities about the knee are one of the common disorders. High Tibial osteotomy is a way of correcting the deformity. Although the general agreement is focused toward the open wedge technique, discussion about the type of device is a subject to debate.

**Objectives:**

This current study has attempted to evaluate the results of Orthopedic Dual Sliding Compression Plate (ODSCP) in high medial open wedge osteotomies of the tibia.

**Patients and Methods:**

In this cross-sectional study, 16 patients with genuvarum undergone high medial tibial open wedge osteotomy and fixed by Orthopedic Dual Sliding Compression Plate. At the time of the last follow up visit, Lysholm score was gathered.

**Results:**

The mean follow-up time was 9.33 ± 1.87 month. The average age was 45.13 ± 7.25 years. Three patients were male and 13 patients were female. The lysholm score showed a significant difference before and after surgery.

**Conclusions:**

The ODSCP has many advantages over the other type of plates. It can help the surgeon to operate with a relaxed mind and it is advisable for high tibial medial open wedge osteotomies.

## 1. Background

Angular deformities about the knee are one of the common disorders, which bring a person to a physician, and the usual complaint is anterior knee pain. Nevertheless, in younger generation the reason for referral can be a cosmetic one. Angular deformities were first described by Mikulicz-Radecki in 1880. He observed that the axis of the lower extremity passes through the three joints of the hip, knee and ankle in normal individuals and the knee center deviates from this line in angular deformities of the knee (1). In this case, the medial compartment will bear the weight in genuvarum and lateral compartment will bear weight in genuvalgum, and osteoarthritis will develop in the mentioned compartment. Since the population in most countries is becoming aged, the genuvarum and consequently the resulting osteoarthritis will increase, and if the patient fulfills the criteria, high tibial osteotomy is advisable (2). High tibial osteotomy was described first by Jackson in 1958 (3) and has nowadays become a well-established surgery. Coventry, who was a pioneer in this field, has published many reports on the results of HTO (3-6). He was applying the lateral close wedge technique for osteotomy, but recently the acceptability of medial open wedge osteotomy is increasing due to lower complications like keeping the proximal tibiofibular joint and peroneal nerve intact. Besides, more accurate correction will be obtained by medial open wedge osteotomy (7). Although the general agreement is focused toward the open wedge technique, discussion about the type of device is a subject to debate, and the devices are under investigation (1, 2, 8-10)

## 2. Objective

This current study has attempted to evaluate the results of Orthopedic Dual Sliding Compression Plate (ODSCP) in high medial open wedge osteotomies of the tibia.

## 3. Patients and Methods

This clinical trial study was done in Ghaem Hospital, Mashhad, Iran. The patients with more than five degrees of knee varus, who had undergone open wedge osteotomies and fixation with ODSCP, were included in this study. The operations were done during September 2011 and February 2012 and 16 patients with 16 knees were assessed. The inclusion criteria were knee varus of more than 5 degrees, knee pain with no response to conservative treatment, appropriate weight and BMI (< 25) and no signs of rheumatoid arthritis. The exclusion criteria were knee flexion of less than 90 degrees, more than 20 degrees of correction needed, lateral tibial subluxation of more than 1cm and three-compartment involvement. The average age of the patients was 45.13 ± 7.25 years and the average time to follow up was 9.33 ± 1.87 month.

### 3.1. Plate Design

**Figure 1.**
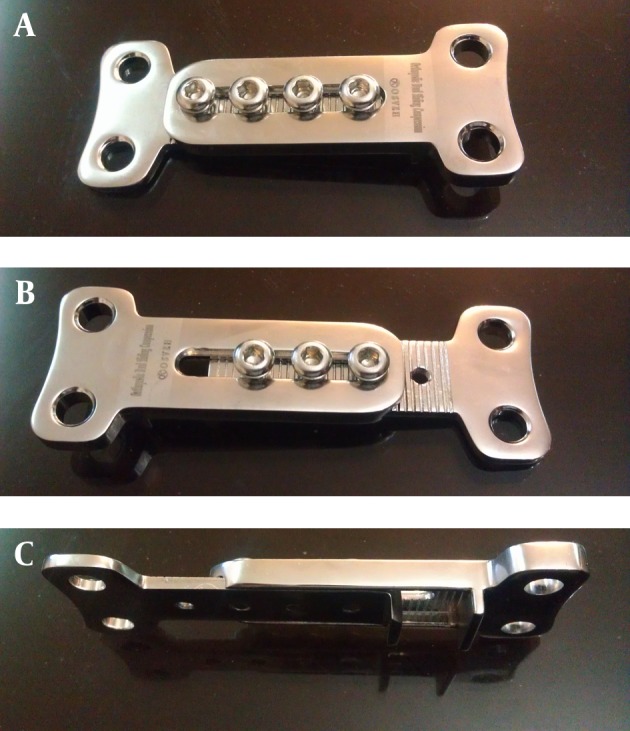
A-C: Design of the Orthopedic Dual Sliding Compression Plate

The alloy of the current devices is steel 316 LVM, which was used to manufacture the ODSCP. This plate is consisted of two separate parts, tongue and buttonhole, which can be joined together like a rail system and can make the length of the plate adjustable (Figure 1 A-C). There are two holes above and two holes below the railing section, which are placed in a horizontal fashion to decrease the length of the plate and the length of the incision as well. The short length is one of the advantages over the other types like T-plate. The proximal part is consisted of two holes for bone screws on one side and the tongue on the other side. There are fine grooves on the anterior side of the tongue and two holes for locking screws. The distal part is consisted of two holes on one side and the buttonhole on the other side. As the locking screws compress the buttonhole on the tongue part, the buttonhole's fine grooves on the posterior side will be joined with the tongue's grooves for stability. Locking screw is the one, which locks the tongue to the buttonhole and is a short screw. The railing design makes up to 2cm lengthening possible. In addition, there is a shelf portion vertically oriented on each part to be placed in the osteotomy groove, sit on the tibial cortex and holds the osteotomy site open. The design can be changed for the number of the holes and the length. So this interchangeability makes this device a modular plate.

### 3.2. Preoperative Assessment

An AP standing hip to ankle radiography (alignment view) was done for the patients before surgery. The calculation of the angle of the deformity was done by drawing the mechanical axis of the femur from the center of the femoral head to the middle of the knee and the mechanical axis of the tibia from the middle of the plateau to the middle of the talar dome. The diameter of the opening wedge was calculated according to the formula W = D*0.02*A. D is the diameter of the tibia at the intended site of osteotomy and A is the angle of deformity which is calculated between the mechanical axes, and is added by 3-5 degrees for overcorrection so that the mechanical axis passes through the 62% of the tibial plateau from the medial side (11). The Lysholm score assessed patients preoperatively and all the patients signed the consent form.

### 3.3. Surgical Technique

**Figure 2.**
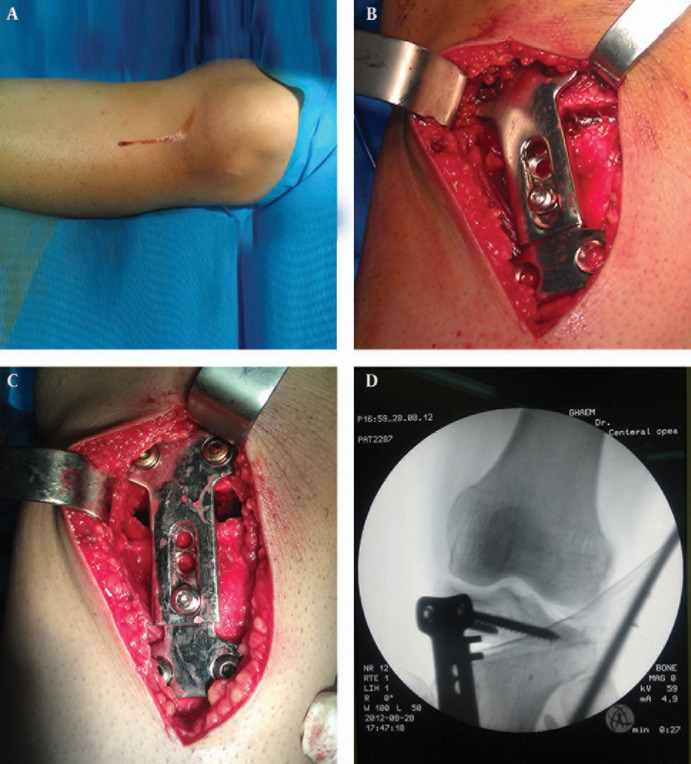
A-D: Surgical Technique for Application of Orthopedic Dual Sliding Compression Plate

The surgery is done on a radiolucent table in a supine position and the pneumatic tourniquet is applied. A vertical incision with a curve portion at the proximal end is placed on the medial proximal tibia and at the level of the tibial tuberosity, which is approximately 6-8cm in length. Fascia and periosteum is incised limitedly in line with the incision. Patellar tendon is released from beneath to be protected and not to be cut during osteotomy. Then two parallel 2mm pins are introduced in the way of the osteotomy from beneath the medial plateau hump and above the tuberosity toward the lateral above the proximal tibiofibular joint. After checking by the fleuroscopy, tibia was osteotomized in line with the guide pins. At this stage, without the need for any distractor, the ODSCP is inserted and screwed on the tibia so that the shelf portion is placed inside the osteotomy site. The proximal screws are cancellous type and distal screws, depending on the quality of the bone, can be cancellous or cortical ones. The locking screws in the middle are loose and the tongue and buttonhole can move along each other easily. Here, the assistant or the distrator can help to open the wedge and correct the varus to the size that was measured preoperatively, and it is check by a ruler at the time of surgery. Meanwhile the locking screws are tightened to compress the grooves of the tongue to the buttonhole. To fill the gap, tricortical bone graft is harvested from the iliac bone and inserted at the entrance of the osteotomy, and the rest of the gap can be filled by chips and cancellous allograft. The subcutaneous tissue and skin are sutured over a closed drain without evacuation (Figure 2 A-D).

### 3.4. Postoperative Assessment and Rehabilitation

Knee immobilizer was used for the patients after surgery and to be worn at nights. Drain was removed after 24 hours, and the patients were discharged the day after surgery. Passive and active knee range of motion and protected tip-toe weight bearing was allowed as tolerated. To decrease the prescription of NSAIDs, we ordered intravenous acetaminophen at the hospital and oral acetaminophen at home. The patient was advised to come back at two weeks for suture removal and to be introduced to a physical therapist to increase the range of motion. The next routine visits were at 6th, 8th and 12th weeks after surgery and any other extra visits could be set if needed. During the visits knee examination and radiography was done. Clinical union was determined according to the absence of pain and tenderness on the palpation, and the radiographic union was determined if the ossified lines were visible in the middle of the gap. In case of clinical union, the patient was allowed to bear weight partially and in case of radiographic union in two planes, the patient was allowed to abandon the crutches and a cane being substituted, if needed. At the time of the last follow up visit, Lysholm score was gathered, radiographic union was assessed and knee was examined for the range of motion and other probable complications. Data was analyzed by SPSS software version 16.

## 4. Results

In this clinical trial study, 16 patients and 16 knees underwent surgery. The mean follow-up time was 9.33 ± 1.87 month. The average age was 45.13 ± 7.25 years. Three patients (18.75%) were male and 13 patients (81.25%) were female. The plate was applied according to the above-mentioned technique. None of the patients involved with nonunion, malunion, compartment syndrome, plate failure, pes anserinus tendinitis and wound infection. The only complaint of two patients was the prominency of the plate and they desired to remove the plate after bone union. When asking if they are ready to be operated on the other knee, 13 patients (81.25%) answered 'yes'. The result is summarized in Table 1.

**Table 1.** The Summaryof Demographic Data and Pre- and Postoperative Assessment of the 16 Patients

Demographic Data	Values
**Age, y, Mean ± SD**	45.13±7.25
**Gender, No. (%)**	
Male	3(18.75)
Female	13(81.25)
**Average follow up time, months, Mean ± SD**	9.33±1.87
**Average time to union, weeks, Mean ± SD**	12.67±1.95
**Operation time, min, Mean ± SD**	21.27±4.63
**Length of incision, cm, Mean ± SD**	7.6±0.82
**ROM, Mean ± SD**	
Before surgery	138±9.96
After surgery	37±8.82
P value	0.271
**Varus angle, Mean ± SD**	
Before surgery	9.8±2.42
After surgery	0.2±1.20
P value	< 0.001^a^
**Lysholm score, Mean ± SD**	
Before surgery	42.33±3.99
After surgery	73.93±4.18
P value	< 0.001

^a^P < 0.05 is considered significant

## 5. Discussion

Patients with varus deformity in their knees are advised to be undergoing corrective surgery as soon as possible (1, 12, 13). There are two techniques of open and close wedge osteotomies. There is a tendency toward the open wedge osteotomy since it has fewer complications on the proximal tibiofibular joint and peroneal nerve. In addition, the difficulties of total knee replacement after close wedge technique are absent (14, 15). The main idea in most recent reports is about the type of the device with fewer complications and more advantages (1, 2, 8-10). In the current study, we tried to evaluate the advantages and disadvantages of the sliding plate. To the best of our knowledge, this current study is the first study, which introduces the innovative sliding plate system for high tibial osteotomy. Staubli reported the results of a specific angular stable plate, Tomofix, in high tibial open wedge osteotomies (1). The plate was 115mm long and was designed for a 10 degrees wedge opening. He evaluated 53 patients with an average age of 50 years, and the mean opening wedge was 5-20 degrees on the medial side. However, the incision was as long as the plate length, and since more screws were needed, the time of surgery would be increased (1). In a study in China on 18 patients, locking compression plate system was applied which had similar features as Tomofix (10). The average time to union was 12-16 weeks and no case of nonunion, malunion, nerve injury or plate failure was observed. Full weight bearing was started after 6-8 weeks. The mean correction of the deformity was 5.5-18 degrees. In the results of their technique, there was no difference in ROM before and after surgery, but the Lysholm score was improved significantly (10). In another study in Korea, Aescula open wedge plate was applied for fixation of the osteotomy site (2). The average time to clinical and radiographic union was 12.69 ± 1.5 weeks, and the incision length was 6cm. The radiographic measurements were improved significantly. In order to fill the osteotomy gap, they used an allograft and then a cylinder cast was applied for 3-4 weeks. No case of nonunion, malunion, compartment syndrome or nerve injury was reported (2). Likewise, in a study in Turkey, they reported the results of applying Pudu Plate, and the results were acceptable (16). Our study consisted of 16 patients with an average varus deformity of 9.8 ± 2.42 degrees who undergone high tibial medial open wedge osteotomy (HTMOWO) and fixation with ODSCP. The average time to union was 12.67 ± 1.95 weeks. The important point in designing this plate is its versatility and adjustability since the two components have a sliding feature and they can be modular. The other advantages of ODSCP over the other types are as follows; first, the short length of the plate needs short incision, which is important for cosmetic reasons. Second, more precise wedge can be opened according to the pre- and perioperative measurement. Third, in the previously mentioned reports, the distractor should be placed first to open the wedge and the plate should be inserted beside the distractor while it holds the wedge open which makes it difficult in a small space and the plate position follows the distractor position. This may lead to correction failure. However, with ODSCP, the plate is inserted at first and if needed the distractor can be placed. Otherwise, the gap can be opened manually. Forth, there is a risk for plateau fracture and extension of the osteotomy line into the articular surface if distractor be placed and forced toward the above side (16, 17). In applying ODSCP, the plate with long cancellous screws are inserted beneath the plateaus at first which can act as a lever for the whole surface of tibial plateau and moves it as one piece during distraction. Fifth, since the plate is inserted before distraction, the potential risk of translation or rotation will be omitted. Sixth, the time of the surgery is short due to the straightforward technique. Seventh, if any mistake in angle correction was detected after plate fixation, there is no need to remove the bone screws in order to correct the error. The only thing is needed is to loosen the small locking screws and adjust the sliding system again. According to the above-mentioned advantages, the ODSCP can help the surgeon to operate with a relaxed mind and it is advisable for high tibial medial open wedge osteotomies. However, we suggest longer follow up studies with larger number of patients to compare different devices and techniques.
